# Normal and tumor-derived organoids as a drug screening platform for tumor-specific drug vulnerabilities

**DOI:** 10.1016/j.xpro.2021.101079

**Published:** 2022-01-10

**Authors:** Camilla Calandrini, Jarno Drost

**Affiliations:** 1 Princess Máxima Center for Pediatric Oncology, Heidelberglaan 25, 3584 CS Utrecht, the Netherlands; 2 Oncode Institute, Heidelberglaan 25, 3584 CS Utrecht, the Netherlands

**Keywords:** Cell Biology, Cancer, Health Sciences, High Throughput Screening, Stem Cells, Organoids

## Abstract

Patient-derived tumor organoids can be predictive of patient’s treatment responses, and normal tissue-derived organoids allow for drug toxicity testing. Combining both types of organoids therefore enables screening for tumor-specific drug vulnerabilities. Here, we provide a detailed protocol for organoid drug screening using, as proof-of-principle, patient-derived malignant rhabdoid tumor organoids. The protocol can be adapted for drug testing on any tumor and/or normal tissue-derived organoid culture.

For complete details on the use and execution of this protocol, please refer to Calandrini et al. (2021).

## Highlights

• Protocol for patient-derived organoid drug screening
• Applicable to both normal and tumor-derived organoids
• Allows for the identification of tumor-specific drug vulnerabilities

## Graphical abstract

**Figure:**
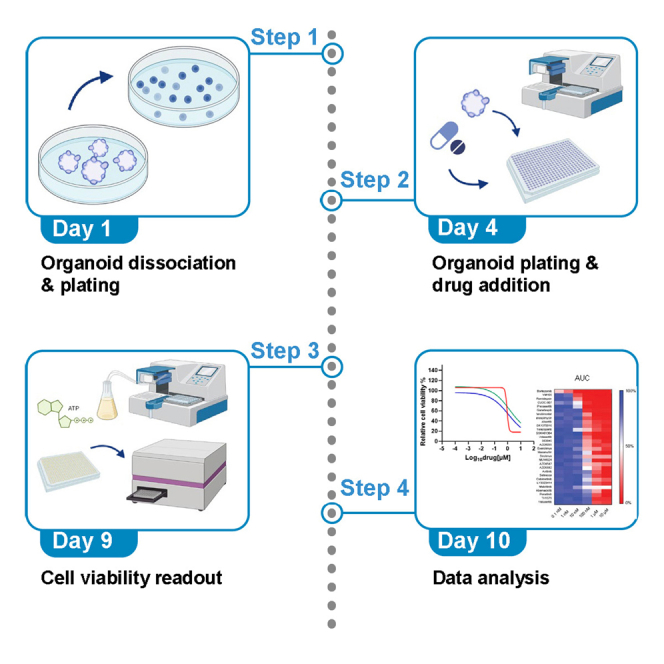

## Teaser

Patient-derived tumor organoids can be predictive of a patient’s treatment response; normal tissue-derived organoids allow for drug toxicity testing. Combining both types of organoids enables screening for tumor-specific drug vulnerabilities. Here, we provide a detailed protocol for organoid drug screening with patient-derived malignant rhabdoid tumors. The protocol can be adapted for high-throughput drug testing on any tumor and/or normal tissue-derived organoid culture.

## Before you begin

**Figure 1:**
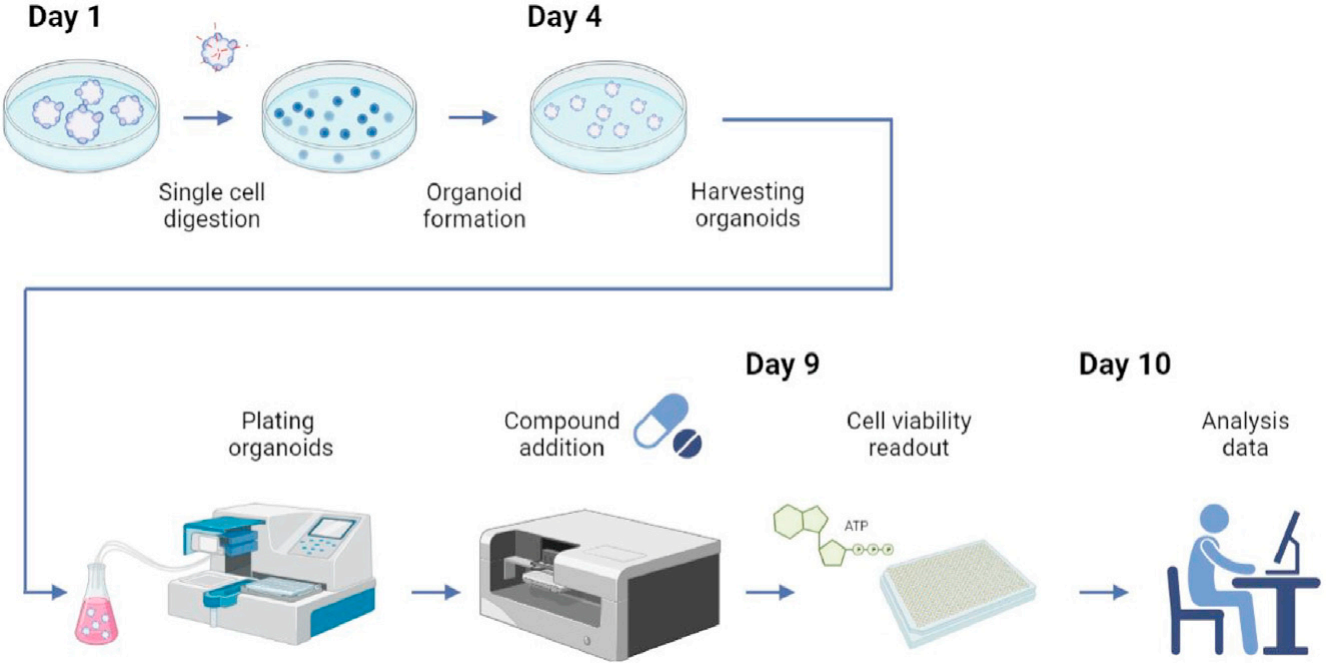
A schematic overview of the protocol

The protocol below describes in detail how to perform drug screens on organoid cultures (Figure 1). For example, we use patient-derived malignant rhabdoid tumor (MRT) organoid cultures (Calandrini et al., 2020, 2021; Custers et al., 2021). Yet, the protocol can be easily adapted to any type of tumor and/or normal tissue-derived organoid culture model.

### Genetic characterization of tumor organoids before drug screening

**Timing: 1–4 weeks**

1. **Verification tumor organoid cultures**: It is crucial to verify the cancerous nature of the established organoid cultures before performing any drug screening experiments. This can be done in different ways, including:
  a. Targeted PCR amplification and subsequent Sanger sequencing of any known tumor-specific somatic mutations identified in the parental tissue;
  b. SNP array to confirm the presence of the same copy number alterations in organoid and parental tissue;
  c. Whole exome/genome sequencing of the organoid culture and subsequent comparison to the genetic profiles of parental tissue.

***Note:*** In case no genetic information is available for the tumor tissue, immunohistochemistry analysis of known tumor markers could be performed on tissue and organoid samples. For instance, in case of MRT, INI1 staining should be performed to confirm lack of expression in tumor cells (Sigauke et al., 2006).

### Preparation screening: Layout screening plate, expansion organoid cultures, preparation reagents and equipment

**Timing: 1–4 months**

2. **Preparation plate layout:** Prepare a plating layout, specifying how many compounds and concentrations will be tested. This is crucial to assess the number of organoids required for the drug screen experiment (see *step 3*). In this protocol, we describe how to perform a drug screen in a 384-well plate layout, with an optimal plating volume of 40 μL per well.
  a. We recommend to test a broad range of concentrations for each compound in order to capture a full dose response curve.
    ***Note:*** this can be achieved by using dilutions ranging from 10 pM up to 10–100 μM. For compounds in clinical development, a literature search can be done to find the most optimal concentration ranges.
  b. The inclusion of a positive and a negative/vehicle control is highly recommended.
    ***Note:*** The vehicle control is required to set the 100% viability threshold at the day of the read-out and to calculate the effect of increasing concentrations of the drug on organoid viability. As vehicle control, the solvent used to dissolve the drug should be used (e.g. dimethylsulfoxide (DMSO)).
    **CRITICAL:** it is important to test the maximum volume of vehicle that can be added to the organoids without affecting cell viability. For instance, we recommend keeping DMSO volumes below 0.5% vol/vol.
  c. When designing the plating layout, ensure that the same final vehicle volume is present in all the wells included in the experiment.
  d. When designing a drug screening experiment, test every concentration with a minimum of three technical replicates.
  e. Randomize the position of the triplicates to ensure that no bias is induced due to location in which the cells are plated within the 384-well plate.
  f. To prevent edge effects, do not plate cells in the outer wells of the screening plate. Instead, plate PBS.
    **CRITICAL:** evaporation of the medium during the drug screening procedure can interfere with the accuracy of the cell viability readout. To prevent evaporation, fill all empty wells with PBS or adDMEM/F12 +++. Dispense 40 μL volume per well. If evaporation is considerable, higher volumes of PBS (up to 80 μL) should be considered.
  g. Include wells with medium only (e.g., adDMEM/F12 +++) in the plate layout to be able to determine the background signal on the day of the readout.

3. **Expansion of tumor organoid cultures:** To proceed with the drug screening, a sufficient number of organoids is required. The number of organoids required depends on:
  a. The growth characteristics of the organoid culture, such as proliferation rate and organoid appearance (i.e., number of cells per organoid);
  b. The number of drugs to be tested;
  c. The number of different concentrations to be tested per drug.
4. **Organoid growth curve**: Before performing the drug screen, an organoid growth curve should be performed to identify the number of organoids needed to achieve the optimal viability at the endpoint of the experiment (maximum viability value before reaching growth curve plateau phase). As indication, seed between 250 to 1000 organoids per well.

***Note:*** This protocol describes the procedure for drug testing in organoids. As such, we plate intact organoid structures and not single cells. In the specific case of MRT cultures, we plate 500 organoids with an approximate size of 3–6 cells per organoid per well.

5. **Calibration tubing:** When using an automated system for organoid dispensing, it is crucial to calibrate the equipment before every drug screen to achieve an accurate plating. We refer to the manufacturer’s instructions for further details about calibration.
6. **Preparation Milli Q, Ethanol 70%, and PBS:** Aliquot Milli Q water, Ethanol 70% (vol/vol) and PBS into 50 mL tubes. For each line that will be plated, prepare a total of ∼500 mL Milli Q, 250 mL Ethanol 70% vol/vol and 50 mL PBS. Store the aliquots at 4°C for a minimum of 12 h.

## Key resources table

**Table:** 

REAGENT or RESOURCE	SOURCE	IDENTIFIER
**Biological samples**		

Patient-derived organoid line 78T	Calandrini et al. (2020)	N/A
Patient-derived organoid line 33T	Calandrini et al. (2021)	N/A
Patient-derived organoid line 60M	Calandrini et al. (2020)	N/A
Patient-derived organoid line 103T	Calandrini et al. (2020)	N/A
Patient-derived organoid line 60H	Calandrini et al. (2020)	N/A
Patient-derived organoid line 103H	Calandrini et al. (2020)	N/A
Patient-derived organoid line 78H	Calandrini et al. (2020)	N/A

**Chemicals, peptides, and recombinant proteins**		

Advanced DMEM/F12	Thermo Fisher Scientific	Cat# 12634010
Glutamax	Gibco	Cat# 35050061
Hepes	Gibco	Cat# 15630106
Penicillin/Streptomycin	Gibco	Cat# 15140163
B27 supplement	Thermo Fisher Scientific	Cat# 17504044
R-spondin conditioned medium	In house production	N/A
N-acetylcysteine	Sigma-Aldrich	Cat# A9165
Primocin	InvivoGen	Cat# ant-pm-1
RhoKinase inhibitor Y-27632	AbMole BioScience	Cat# M1817
EGF	PeproTech	Cat# AF-100-15
FGF10	PeproTech	Cat# 100-26
A83-01	Tocris Bioscience	Cat# 2939/10
BME	Trevigen	Cat# 3533-010-02
Cell Recovery Solution	Corning	Cat# 354253
Trypan blue Solution	Thermo Fisher Scientific	Cat#15250061
TrypLe Express	Thermo Fisher Scientific	Cat# 12605010

**Critical commercial assays**		

CellTiter-Glo 3D reagent	Promega	Cat# G9683

**Software and algorithms**		

GraphPad Prism v7.04	GraphPad software	https://www.graphpad.com/
Tecan D300e	Tecan software	https://www.tecan.com/

**Other**		

384 well plates black/flat bottom	Corning	Cat# 3542
12 well tissue culture plates	Greiner Bio-One	Cat# 665180
6 well tissue culture plates	Greiner Bio-One	Cat# 657160
70μm cell strainer	Greiner Bio-One	Cat# 542070
15 mL tubes	Falcon	Cat# 10773501
50 mL tubes	Falcon	Cat# 10788561
Medium bottle, 125 mL	Nalgene	Cat# C483.1
Hemocytometer	Marienfeld	Cat# 0640211
Multidrop Combi Reagent dispenser	Thermo Fisher Scientific	Cat# 5840300
Multidrop Combi Reagent dispenser tubing set	Thermo Fisher Scientific	Cat# 24072670
Automated liquid handler Tecan digital dispenser D300e	HP	https://www.tecan.com/
Dispensing cassettes small volume (T8+)	HP	Cat# 30097370
Dispensing cassettes high volume (D4+)	HP	Cat# 30097371
Luminescence plate reader FLUOstar Omega reader	BMG LABTECH	https://www.bmglabtech.com/fluostar-omega/

## Materials and equipment

**Table:** AdDMEM/F12 +++ medium

Reagent	Final concentration	Amount
Hepes	10 mM	5 mL
Glutamax	1×	5 mL
Penicillin/Streptomycin	100 U/mL	5 mL
Advanced DMEM/F12		500 mL
**Total**		**515 mL**

Store AdDMEM/F12 +++ medium at 4°C for up to 4 weeks.

### Organoid medium for MRT growth

The organoid medium for MRT growth was established by Schutgens et al. (2019), adapted from the medium originally developed for normal kidney tubuloids growth (Schutgens et al., 2019).

**Table:** 

Reagent	Final concentration	Amount
A83-01	5 μM	50 μL
B27 supplement	1.5%	750 μL
Human EGF	50 ng/mL	5 μL
Human FGF10	100 ng/mL	50 μL
N-acetylcysteine	1.25 mM	125 μL
Primocin	0.1 mg/mL	50 μL
RhoKinase inhibitor Y-27632	10 μM	50 μL
R-spondin conditioned medium	10%	5 mL
AdDMEM/F12 +++		43.92 mL
**Total**		**50 mL**

Store organoid medium at 4°C for up to 3 weeks.

## Step-by-step method details

### Single cell dissociation and plating of organoids

#### Day 1

**Timing: 1 h (per organoid line)**

This section describes the experimental steps to expand and plate organoids for drug screening. We use MRT organoids as example, but in principle the same steps can be applied to any type of organoid model.

1. Collect the organoids embedded in a BME droplet with a p1000 pipette using the culture medium in the well and transfer to a 15 mL tube.
2. Pipette up and down to disrupt the BME droplets.
3. Fill up the tube with ice-cold adDMEM/F12 +++.
4. Centrifuge at 300 × *g* for 5 min at 4°C.
5. Remove supernatant.
6. Add pre-warmed (37°C) TrypLE with addition of 10 μM Y-27632 to the pellet. Use 1 mL solution per 200 μL of harvested BME.
7. Incubate for 3–4 min at 37°C.
8. To mechanically dissociate the organoids, pipette up and down for 10–15 times using a fire polished glass pipette.
9. Visually inspect the organoid mixture under a brightfield microscope. At this point, most of the suspension should composed out of single cells.

***Optional:*** if this is not the case, repeat steps 7–9 once more. Troubleshooting 1

10. Fill up the tube with ice-cold adDMEM/F12 +++.
11. Centrifuge at 300 × *g* for 5 min at 4°C.
12. Remove supernatant.
13. Measure the pellet size using a p100/p1000 pipette set to a known volume, or make use of falcon tubes filled with standard volumes to infer the volume of the pellet. Add 70% volume of ice-cold BME to the pellet.

***Optional:*** if organoids tend to stick to the plastic surface of the tip when pipetting, precoat the tip by pipetting up and down with adDMEM/F12 +++.

**Figure 2:**
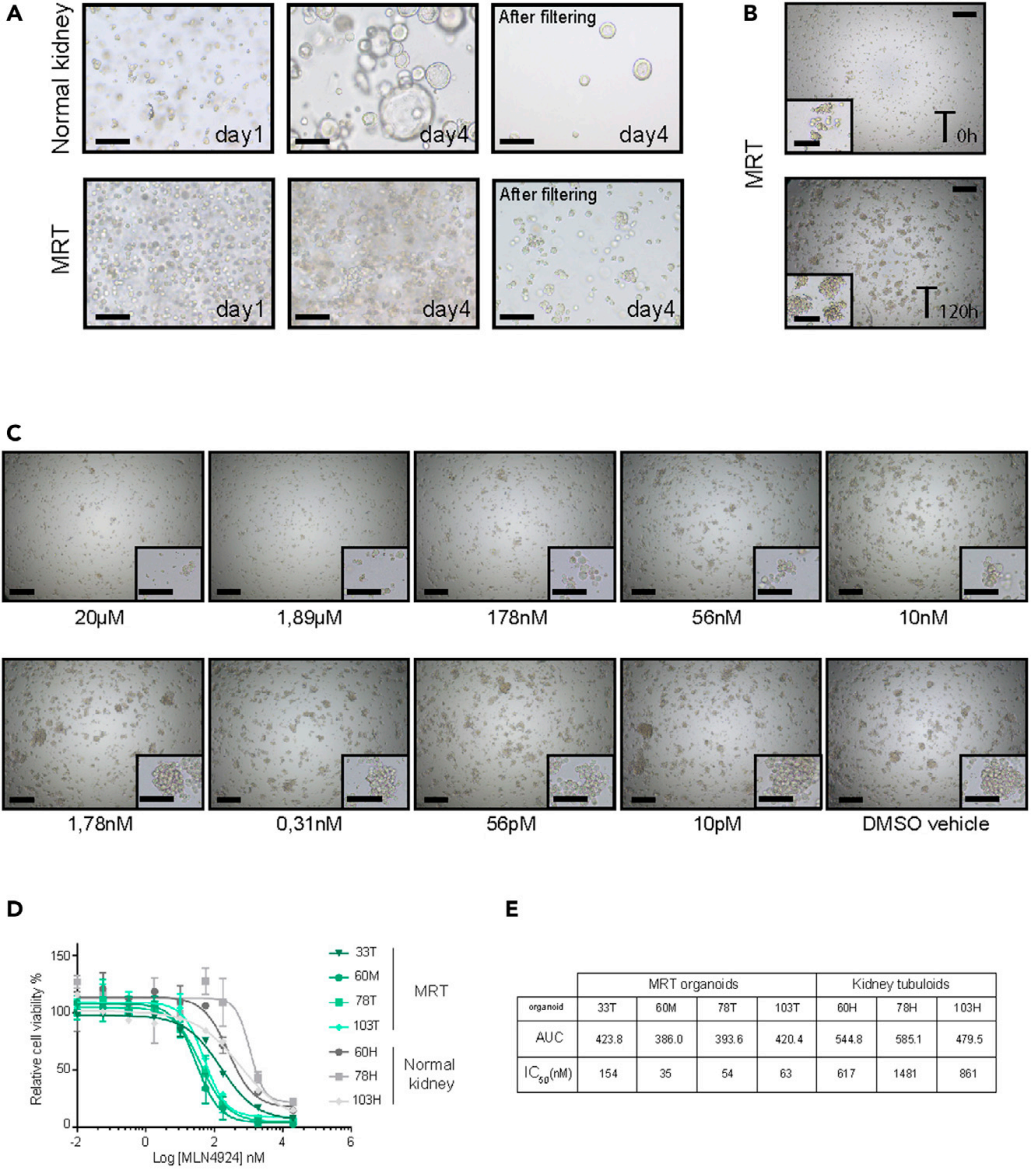
Patient-derived organoids as drug screening platform (A) Representative brightfield images of normal kidney tubuloids (top) and MRT organoids (bottom) at different stages of preparation for drug screening. Sample at single cell state (*step 16*, left), sample prior to harvesting (*step 17*, middle) and sample after filtering (*step 25*, right). Scale bars 200 μm. (B) Representative brightfield images of MRT organoids upon plating in 384-well drug screening plates at timepoint T_0h_ (top) and timepoint T_120h_ (bottom). Scale bars 500 μm, zoom in 200 μm. (C) Brightfield images of MRT organoids plated in 384-well drug screening plates and treated with a range of different MLN4924 concentrations (20 μM–10 pM) for a 5-day period. Scale bars 500 μm, zoom in 200 μm. (D) Example of dose-response curves of MLN4924 for the indicated MRT and normal kidney tubuloid organoid cultures. Error bars represent SD of 2 independent experiment, each consisting of 4 technical replicates. (E) Example of area under the curve (AUC) and inhibitory half concentration (IC_50_) values calculated from the drug screening data presented in (D). Adapted from Calandrini et al. (2021).

14. Carefully resuspend the pellet in the leftover supernatant without creating air bubbles and plate the mixture with droplets of 15–20 μL volume into prewarmed 12 well or 6 well plates.
15. Place the plate in the 37°C incubator upside down for 20–25 min, to prevent adherence of the cells to the plate bottom.
16. Add pre-warmed (37°C) organoid culture medium to the wells and inspect the culture under a brightfield microscope (Figure 2A).

### Preparation of organoids for dispensing into multi-well drug screening plates

#### Day 4

**Timing: 1–2 h (per organoid line)**

This section describes the harvesting, counting and preparation of the organoid solutions for dispensing into multi-well drug screening plates. We use MRT organoids as example, but the basic principles can be generally applied to any type of organoid model.

17. Visually inspect the organoid cultures using a brightfield microscope to assess their viability and size. As example, MRT organoids should be at the 3–6 cells stage. (Figure 2A) Troubleshooting 2
18. Collect the organoids embedded in a BME droplet with a p1000 pipette using the culture medium in the well and transfer to a 15 mL tube.
19. Fill up the tube with ice-cold adDMEM/F12 +++.
20. Centrifuge at 300 × *g* for 5 min at 4°C.
21. Remove supernatant.
22. Repeat washing step by adding 10 mL of ice-cold adDMEM/F12 +++ and resuspend the pellet. Troubleshooting 3
23. Centrifuge at 300 × *g* for 5 min at 4°C.
24. Remove supernatant; add 5 mL of adDMEM/F12 +++ and resuspend the pellet.
25. Filter the organoid solution through a 70 μm strainer placed on top of a 50 mL tube. (Figure 2A) Troubleshooting 4
26. Transfer the filtered solution to a 15 mL tube.
27. Centrifuge at 300 × *g* for 5 min at 4°C.
28. Remove supernatant and resuspend pellet in a total volume of 1 mL, making use of ice-cold adDMEM/F12 +++ to achieve the correct volume.

**CRITICAL:** The organoid suspension must be thoroughly mixed to obtain a homogeneous solution that is devoid of cell clumps. This step is critical for an as accurate as possible estimation of the number of organoids in solution.

29. Take 1–5 μL of the organoid mix and count the number of organoids using a hemocytometer.

***Note:*** each organoid is count as a single unit.

30. Calculate how many organoids are present in 1 mL. If the number of organoids is sufficient, transfer the required volume to a new tube and proceed with protocol. Otherwise, re-plate the organoids as described in steps 10–16 to further expand the culture.

***Note:*** The total number of organoids needed depends on the number of wells required for the experiment. In the case of a 384-well plate set-up, plate 500 organoids per well (in total ∼160,000 organoids per 384-well plate).

31. Prepare the organoid solution by mixing organoids, organoid culture medium and 5% vol/vol BME matrix in a 50 mL tube or a sterile flask. Mix by inversion - preventing air bubbles - and put on ice.

***Note:*** The total volume required depends on the number of wells that are used for the experiment. In a 384-well plate set-up, dispense a total volume of 40 μL per well, for a total of ∼ 13 mL volume per screening plate. Calculate extra volume to compensate for plating inaccuracy/dead volume needed for dispensing. We recommend calculating at least 10% extra volume/organoids per screen.

### Dispensing organoid mixture into 384-well drug screening plates

#### Day 4

**Timing: 30 min (per organoid line)**

The following steps describe the plating of the organoid solution into 384-well screening plates. In order to plate the organoids in the most accurate manner make use of a reagent dispenser machine (e.g., Multidrop Combi Reagent dispenser (ThermoFisher)). The reagent dispenser should be placed on a cleaned bench or in a flow hood to ensure a sterile environment. Note that all solutions should be ice-cold to prevent polymerization and solidification of the BME in the tubing.

32. Clean, disinfect and cool down the tubing of the reagent dispenser by flushing with 100 mL (2× 50 mL tube) of Milli Q water, followed by flushing with 100 mL of 70% vol/vol Ethanol, and finally once more with 100 mL of Milli Q water.
33. Wash the tubing with 25 mL PBS. To avoid medium evaporation during the experiment, dispense 40 μL of PBS solution to the wells of the 1^st^ and 24^th^ column (outer wells of the multi-well plate).
34. Prepare the tubing for organoid solution dispensation by flushing with 25 mL of ice-cold adDMEM/F12 +++. Subsequently, plate 40 μL of adDMEM/F12 +++ solution to the wells of the 2^nd^ and 23^rd^ column, as well as to the top and bottom rows on the multi-well plate.
35. Before dispensing, gently invert the organoid-containing solution to ensure that the mixture is as homogenous as possible.
36. Empty the tubing and prime the organoid mixture until the solution has filled every capillary of the tubing.

**CRITICAL:** Make sure that the tubing is ice-cold before priming the organoid mixture. Proceed promptly to avoid the tubing from reaching 15°C–18°C and the BME to solidify.

37. Proceed with the plating of the organoid mixture to the drug screening plate by dispensing 40 μL of organoid mixture to the selected columns/wells.

**CRITICAL:** To ensure a homogeneous distribution of the organoids during dispensing, gently swirl the solution while plating. Make sure not to generate air bubbles by swirling too vigorously.

38. In a separate screening plate, dispense the organoid mixture in several (at least 3) wells to measure cell viability at timepoint 0 (T_0_). (Figure 2B) This is described in more detail in steps 47–53.
39. Directly after finishing with dispensing the organoid mixture, empty the tubing and prime Milli Q water to start the cleaning procedure.

**CRITICAL:** do not leave the tubing primed with organoid mixture to avoid clogging due to BME polymerization and/or precipitation of reagents.

40. Continue with the cleaning procedure by flushing the tubing with 100 mL (2× 50 mL tubes) Milli Q water, followed by 100 mL of 70% vol/vol Ethanol and again 100 mL of Milli Q water.
41. Visually inspect all plates to confirm that the organoids are plated homogeneously throughout the different wells and plates.
42. Place the screening plates in an incubator at 37°C. Wait at least 4 h before proceeding with the addition of the drugs.

***Optional:*** Use leftover organoids for genomic DNA extraction and subsequent genotyping applications.

### Compound addition—Low throughput

#### Day 4

**Timing: 30 min**

This section describes the addition of the compounds to the screening plates. Based on the number of compounds and concentrations to test, different automated systems for drug dispensing can be used. Here, we describe the addition of compounds for a low throughput screening using the Tecan D300e Digital Dispenser (HP). The machine should be used in a clean bench set up, preferably positioned in a chemical fume hood.

43. Thaw the drug aliquots. Prepare intermediate drug dilutions, if required.
44. In the Tecan D300e software, open the plating layout previously prepared (see section “before you begin”, step 2).
45. Add the compounds and vehicle fluids to the wells following the Tecan D300e software instructions.
46. Place plates back into the incubator at 37°C.

### Readout cell viability with Cell Titer Glo 3D

#### Day 9

**Timing: 45 min (per multi-well plate)**

Here, we describe the procedure for cell viability measurements using Cell Titer Glo 3D (Promega). In this protocol, the readout is performed at 2 timepoints, T_0_ (day of plating) and T_120h_ (5 days, endpoint experiment).

47. Make sure the Cell Titer Glo 3D (Promega) and the multi-well screening plates are at 21°C before starting.
48. Visually inspect plates to ensure that the organoids in the wells containing the vehicle control have grown as expected. (Figures 2B and 2C) Troubleshooting 5
49. Prepare and clean the tubing of the multidrop Combi as illustrated in step 32.

**CRITICAL:** Use solutions stored at 16°C–24°C since temperature fluctuations can interfere with Cell Titer Glo 3D luminescence readout.

50. Use the multidrop combi system to dispense 40 μL of Cell Titer Glo 3D (1:1) in each well.

**CRITICAL:** During dispensing, protect the tubing from light exposure by covering with aluminum foil.

51. Use a horizontal shaker to agitate the multi-well plate(s) for 5 min at 600 rpm.

**CRITICAL:** Prior to shaking, wipe the lid of the plate from any condensed water, as this may cause bubble formation and interference with the accuracy of the readout.

52. Incubate the multi-well plate(s) covered with aluminum foil for 25 min at 16°C–24°C.
53. Measure the luminescence signal per well using a suitable reader (e.g., FLUOstar Omega, BMG Labtech).

## Expected outcomes

This protocol allows for drug testing of any patient-derived organoid culture in a low- to medium-throughput manner. If effective, a visible reduction in cell growth compared to vehicle control should be detectable after 120 h incubation with the compounds of interest (Figure 2C).

After the readout, dose response curve can be generated and parameters such as Area Under the dose response Curve (AUC) and inhibitory half concentration (IC_50_) values can be calculated (Figure 2D,E). This set-up can be used to identify tumor-specific and/or patient-specific therapeutic vulnerabilities, as described (Calandrini et al., 2020, 2021; Custers et al., 2021; Driehuis et al., 2019; Kim et al., 2019).

## Quantification and statistical analysis

In order to convert raw luminescence data to dose-response curves, one can make use of Microsoft Excel and/or GraphPad Prism software.

1. Open the raw data in Microsoft Excel.
2. Retrieve the information of the plating layout to determine which value corresponds to which sample.
3. Calculate the average background luminescence value using the values of the wells containing only medium (no cells (i.e., 0% viability value)). Subtract the average background value from the values of the other wells.
4. Calculate the average luminescence value of the vehicle control wells. This value will be set as 100% viability. Troubleshooting 6
5. Calculate relative cell viability percentage of the other wells using the following equation:

Viabilitywell=(luminescencevaluewellluminescencevehiclecontrol)∗100%

6. Transfer the relative cell viability values calculated and corresponding drug concentration - converted in logarithm values - to GraphPad Prism, under the XY table option.
7. Draw dose response fitting curves by selecting *New analysis* → *Nonlinear regression curve* → *log(inhibitor) vs response – variable slope.* Troubleshooting 7
8. In the resulting table, you will find the calculated IC_50_ value.
9. Calculate AUC values making use of the option *Area under curve* under the tab *Analyze.*
10. To evaluate growth ratios of the organoid lines, compare luminescence values measured at timepoint T_0_ with those measured at T_120h_, using the following equation:

Growhratio=(luminescencevalueuntreatedwellT120luminescencevalueuntreatedwellT0)

## Limitations

In order to perform drug screening in a low to medium-throughput fashion, a significant number of organoids are required. Depending on the type of organoid model, this expansion step can range from several weeks to sometimes even months after establishment of the culture from primary material. As a consequence, drug screening in some cases is only possible several months after culture establishment (Drost and Clevers, 2018; Veninga and Voest, 2021). Although it remains unclear whether prolonged organoid culturing results in the enrichment of specific tumor subclones, we recommend performing any type of drug testing assay on early passage cultures.

Cultures conditions used for growing tumor organoids were often optimized for the growth of normal tissues (Drost and Clevers, 2018). As a consequence, the presence of normal cells in the tumor resection specimen can result in overgrowth by normal organoids (Dijkstra et al., 2020). This can be prevented by using culture conditions that selectively support tumor cells growth. For instance, in the case of TP53-mutated tumors, the P53 stabilizing agent Nutlin-3a can be added to the culture medium (Drost et al., 2015; Calandrini et al., 2020). When considering the case of MRT of the kidney – P53 wild type tumors of mesenchymal origin, characterized by a lack of epithelial differentiation (Young et al., 2021; Judkins et al., 2004) - MRT organoids can be separated from contaminating normal kidney epithelial cells based on the negativity for epithelial markers, such as EPCAM (Calandrini et al., 2020). When these strategies are not possible, it is important to ensure that the tissue specimen is not contaminated with normal tissue. For instance, part of the obtained specimen can be used for histological examination. In any case, it should be verified that the organoid culture is still representative of the parental (tumor) tissue prior to drug screening (see characterization of tumor organoid cultures). Furthermore, although organoids have been shown to be valid models that recapitulate features of the tissue they were derived from, the lack of vasculature, tumor microenvironment and immune cells are still important limitations to be considered (Ooms et al., 2020; Veninga and Voest, 2021; Drost and Clevers, 2018), which currently still limits the use of organoids for immunotherapy testing.

Here, we describe the use of patient-derived organoids to test drug efficacy in a 120 h (5 day) time window. The here-described timelines and operations can be adjusted based on (1) the growth speed of the cultures and, as a consequence, the potential need for medium refreshing and (2) the stability of the tested compounds. If longer treatment periods are required, adjustments to the protocol – such as medium refreshing, organoid passaging, and compound re-addition – have to be considered.

The Cell Titer Glo 3D assay is used in this protocol to infer the relative cell viability from the metabolic activity of cells via the quantification of ATP molecules. This readout cannot therefore distinguish between a cytostatic (reduction in growth) and a cytotoxic (cell death) effect of the tested compounds, since both would cause a reduction in ATP production compared to an untreated control. Therefore, to further characterize the drug-induced effects, additional validation experiments such as apoptosis and cell proliferation assays should be performed (Calandrini et al., 2021).

## Troubleshooting

### Problem 1

Intact organoids or pieces of organoids remain after the digestion procedure *(step 9).*

### Potential solution

Carefully increase the incubation time with TrypLE, followed by mechanical disruption (up and down pipetting). Repeat the process for a maximum of 3 times. Stop the procedure as soon as substantial cell lysis (>30%) is detected in the solution. Cell death quantification should be performed via staining with markers/dyes discriminating live and dead cells (e.g., Trypan blue).Note that it is not always possible to fully digest organoids into a single cell suspension without causing significant cell death in the cultures. In such a case, aim for obtaining a suspension that is as homogeneous as possible, performing mechanical and enzymatic disruption until organoids are dissociated into fragments of equal size (< 70 μm). At this point, big organoid fragments still present in solution can be filtered out using a 70 μm strainer. Notably, take the size of the disrupted organoids into consideration when determining the correct timing for organoid harvesting for drug screening (*step 17*).

### Problem 2

Organoids did not reach optimal size for drug screening plating (*step 17*) because (1) the culture is still composed out of single cells or (2) the structures are too big (diameter > 70 μm).

### Potential solution

Wait (>3 days) until the culture has recovered and organoids have reached a sufficient size before harvesting. A longer period of recovery upon single cell digestion could be a sign of a too harsh/not careful handling of the organoids. Optimize the timing of and method for organoid dissociation.

Reschedule the harvesting of the organoids. It is critical to follow the optimized digestion protocol of the organoid lines strictly and consistently, as it will ensure a predictable growth rate of the cultures and make the results reproducible.

### Problem 3

Large amount of BME is present after washing steps (*step 22*).

### Potential solution

When dealing with large amounts of BME (> 800μL), at the moment of harvesting divide the content in to several falcon tubes, so that a maximum of 400 μL BME is present in each tube. If the issue persists, before proceeding with the filtering step, it is recommended to perform a short enzymatic digestion with TrypLE or Cell Recovery Solution.

### Problem 4

Insufficient organoids remain after filtering (*step 25*).

### Potential solution

Loss of organoids during the filtering step can be caused by (1) insufficient digestion of the culture when plating (see problem 2); (2) high content of BME remaining in the organoid solution. When a considerable amount of BME is still visible at step 24 (recognizable as a thick white ring on top of the organoid pellet), wash the organoid pellet once more with ice-cold adDMEM/F12 +++. If after filtering a layer of BME is still present on top of the filter, add 5/10 mL of ice-cold adDMEM/F12 +++ to the strainer and use the tip of a 10mL pipette to scrape the surface of the strainer. At this point, if part of the solution cannot pass through the filter, use a new strainer.

### Problem 5

Insufficient growth in untreated control wells at T_120h_ (*step* 48).

### Potential solution

Possible causes include: (1) inadequate growth factor activity in the culture medium which can be solved by making a new batch of medium; (2) limited viability of the organoids at the moment of plating, which can be solved by using less harsh conditions during digestion and plating of the cultures; (3) the growth of the organoids was reduced due to the different plating set-up (5% BME slurry instead of 70% BME), which can be solved by performing a growth curve replicating the screening conditions prior to the screen, to verify changes in growth rate.

### Problem 6

Large variation in cell viability values between technical replicates (*step* 4).

### Potential solution

This can be caused by an insufficient number of plated organoids per well and/or by a non-homogeneous dispensing of the organoids. Repeat the screen and dispense more organoids per well. If the organoid cultures show a change in growth speed during the expansion phase, we recommend to replicate the growth curve experiment to assess the best organoid seeding density. To avoid a heterogeneous dispensing of the organoids upon plating, ensure that a homogeneous population of organoids is present at the moment of harvesting. Furthermore, be mindful that the organoid solution should be regularly inverted/mixed prior and during dispensing to avoid sinking of the organoids to the bottom of the tube.

### Problem 7

Drug screening results are not reproducible (*step 7*).

### Potential solution

This could be caused by (1) overgrowth of normal cells/ cross-contamination with other lines; (2) low quality/deterioration of drug aliquots. To verify that no cross-contamination has taken place in the organoid cultures, harvest genomic DNA and verify via genotyping/SNP array that the genomic profile is matching those of the early passage cultures and parental tumor tissue. Check the storing conditions and the lifetime/stability of the used compounds; make use of fresh aliquots when repeating the screen.

## Resource availability

### Lead contact

Further information and requests for resources and reagents should be directed to and will be fulfilled by the lead contact, Jarno Drost (J.Drost@prinsesmaximacentrum.nl)

### Materials availability

This study did not generate new unique reagents.

## Data Availability

This paper did not generate new datasets.
